# Management of benign nodular thyroid disease: a nationwide survey of endocrine specialists in Spain

**DOI:** 10.1530/ETJ-24-0313

**Published:** 2025-04-01

**Authors:** Juan J Díez, Juan C Galofré

**Affiliations:** ^1^ Department of Endocrinology, Hospital Universitario Puerta de Hierro Majadahonda, Instituto de Investigación Sanitaria Puerta de Hierro Segovia de Arana, Majadahonda, Spain; ^2^ Department of Medicine, Universidad Autónoma de Madrid, Madrid, Spain; ^3^ SEEN (Sociedad Española de Endocrinología y Nutrición) Thyroid Task-Force, Madrid, Spain; ^4^ Department of Endocrinology, Clínica Universidad de Navarra, Pamplona, Spain; ^5^ IdiSNA (Instituto de investigación en la Salud de Navarra), Pamplona, Spain

**Keywords:** benign, nodular, goitre, management, nodular thyroid disease, survey

## Abstract

**Background:**

Management of benign nodular thyroid disease (BNTD) has changed dramatically over the past two decades, as reflected by international guideline recommendations.

**Purpose:**

We sought to document the preferences regarding the management of euthyroid BNTD among thyroid-dedicated members of the Spanish Society of Endocrinology and Nutrition (SEEN) and assess the extent to which present international guideline recommendations have been incorporated into ordinary practice.

**Methods:**

An online survey on the management of a standard case of BNTD among SEEN thyroid experts and exploration of variations in management following different clinical scenarios, in which variables such as sex, age, ultrasound characteristics, fine needle aspiration results or patient preferences change.

**Results:**

Two hundred and eleven (9% SEEN members) participated in the survey. Most of them, 147 (69.7%), recommended periodic monitoring, 43 (20.3%) surgery and 21 (10.0%) minimally invasive procedures (MIP). No participant opted for levothyroxine or radioiodine. Management of BNTD was modified based on patient preferences, both in favour of more aggressive (surgery) and more conservative (MIP or monitoring) options.

**Conclusions:**

The vast majority of Spanish thyroidologists followed the international guideline recommendations for BNTD management. The trend shows the positive impact of the guideline recommendations with a shift towards more conservative management, taking into account patient preference as a binding element in therapeutic decision-making.

## Introduction

The presence of benign nodular thyroid disease (BNTD) is a very common finding in routine clinical practice (1). Euthyroid BNTD encompasses a wide spectrum of entities, from a thyroid incidentaloma detected by imaging to a thyroid nodule found in a routine medical check-up (2). Usually, BNTD causes mild or no symptoms. Studies on the natural history of BNTD show that two out of ten cases may grow and cause local symptoms (3).

International recommendations for asymptomatic BNTD management generally advise monitoring, although they allow for a range of possibilities (4, 5, 6). Thus, the therapeutic options for growing or symptomatic BNTD remain open. The spectrum includes from continuing monitoring to a more interventionist approach, such as thyrotropin (TSH) suppression or minimally invasive procedures (MIP), or even aggressive possibilities such as radioactive iodine or surgery (4, 5, 6, 7, 8, 9). Each of these different options has advantages and drawbacks and needs to be personalised (10). Nonetheless, the indications for each procedure in the different patient profiles are not clearly defined. The appearance of MIP in the armamentarium and the current criteria on avoiding overtreatment in malignant or suspicious nodules undoubtedly has the potential of influencing therapeutic decisions. At the same time, there are more modulating factors such as patient characteristics (sex, age and comorbidity), physician or patient preferences or the availability of therapies, which determine the choice of treatment (6).

However, despite these broad therapeutic options, international guidelines unanimously advise against treatment with levothyroxine to suppress TSH (4, 5, 6). Notwithstanding this clear recommendation, in the Spanish national THESIS (Treatment of Hypothyroidism in Europe by Specialists: An International Survey) survey, we unexpectedly found that 21.2% national endocrinologists stated that simple growing goitre was an indication for treatment with levothyroxine (11). This proportion is even greater in other European countries, according to data collected in the THESIS aggregate paper on goitre (12). The Spanish THESIS survey (11) was part of an ambitious pan-European survey on the use of thyroid hormones. In that study, we aimed to assess the current attitudes of Spanish members of the SEEN towards the conventional treatment of hypothyroid patients with LT4 and the non-conventional use of LT4 in euthyroid subjects in their daily clinical practice.

Regardless of the international recommendations for the management of BNTD, there is very little information on how specialists deal with it in real life. A previous study in Spain showed wide variability in clinical practice (13). For these reasons, we designed a national survey with the aim of shedding light on euthyroid BNTD management in real clinical practice by endocrinologists with special dedication to thyroid disease. With this information, we aimed to document the preferences regarding the management of euthyroid BNTD among endocrinologists in our country and explore associations with respondents and different clinical situations.

## Methods

### Scope of the study

The scope of this study was made up of endocrinologists with professional experience in thyroid diseases and members of the Spanish Society of Endocrinology and Nutrition (Sociedad Española de Endocrinología y Nutrición, SEEN).

### Survey design

We developed a questionnaire to be answered anonymously online. The questionnaire collected information regarding the demographic and professional data of participants and their preferences for management in a standard patient with euthyroid BNTD and in several clinical variants. Five management modalities were considered: monitoring, levothyroxine, surgery, radioiodine and MIP.

The standard patient was a 50-year-old woman with a single, solid, well-defined thyroid nodule, 4 cm in size, without extrathyroidal extension, and sonographically classified as intermediate risk or moderately suspicious (TIRADS 4). The patient was asymptomatic and her thyroid function tests were normal. The fine needle aspiration (FNA) cytological study was benign.

The clinical alternatives included variations according to the characteristics of the BNTD (nodule size, multinodular goitre, cystic nodule, visible with the head in a normal position, endothoracic component, nodule detected in positron emission tomography/computed tomography (PET-CT), low risk and high risk), the patient characteristics (age, male sex, comorbidity that does not contraindicate surgery, family history of non-medullary thyroid cancer, personal history of cervical irradiation in childhood and oncological disease), or the patient’s own preferences regarding available treatments. A question was also raised about whether treatment would be differed if the doctor were the patient.

Thereafter, we asked the interviewees’ opinions about the complementary tests and their frequency during BNTD follow-up, the criteria for repeating the FNA and the time to discharge the patient. Our last question was under what clinical situation(s) they would change the initial recommendation to either monitor patients with BNTD or to treat them with levothyroxine. In this question, participants were informed that growth at follow-up meant that during follow-up, an increase of more than 20% in the diameter of the dominant nodule (with a minimum of 3 mm) had been observed. It was also specified that the appearance of symptoms at follow-up meant that during follow-up, symptoms appeared that the patient had not previously had, such as dysphonia, dysphagia, dyspnea, pain or clinically relevant local discomfort.

### Ethical approval

This survey received a favourable report from the Research Ethics Committee of the Hospital Universitario Puerta de Hierro Majadahonda (code 57/899280.9/23). Furthermore, before its diffusion, the boards of directors of SEEN and its Thyroid Task Force Steering Committee approved the study. The participants were not asked for any personal data that could make their identification possible to ensure anonymity.

### Dissemination and data collection

The survey was posted on the SEEN webpage, available to all members (*n* = 2,383) from March 1 to Jun 15, 2024. Invitation emails were sent to members immediately before initiating the survey. Two reminder mailings were subsequently sent, one in April and another in May. Participant responses were electronically compiled, saved and hosted on an open-access form creation website (https://www.google.com/forms/). Only the authors had access to the survey database.

### Statistical analysis

Quantitative variables are expressed as median (interquartile range, IQR). For comparisons of means between two groups of subjects, the Mann–Whitney U test was used. Categorical variables are described as absolute values and percentages. Chi-square tests and Fisher’s exact tests were used to compare proportions (unpaired data). All used tests were two-sided, and differences were considered significant when *P* < 0.05.

## Results

### Surveyed endocrinologists

We obtained 211 responses, representing 8.9% of the 2,383 SEEN members, but approximately double the members of the SEEN Thyroid Task Force (which was made up of 124 members). Most responders were women (62.1%) with a median age of 46 years and a median time of professional practice of 20 years. More than two-thirds of surveyed physicians (70.0%) worked in the public health system and had high professional expertise in BNTD (the proportion of patients in their clinics with BNTD was greater than 30% in 74.0% of them). The rest of the professional characteristics are shown in Table 1.

**Table 1:** Demographic and professional characteristics of the 211 interviewed endocrinologists. Data are presented as *n* (%) or as median (IQR).

Characteristics	Values
Sex	
Female	131 (62.1)
Male	80 (37.9)
Age, years	46 (38–56)
Time in medical practice, years	20 (11–30)
Non-assistance activities	
Teaching	168 (79.6)
Research	112 (53.1)
Management	45 (21.3)
Membership	
European	42 (19.9)
International	30 (14.2)
Place of employment	
Public	149 (70.6)
Private	23 (10.9)
Public and private	39 (18.5)
Proportion of patients with NTD	
≤30%	55 (26.1)
31–60%	140 (66.4)
61% or more	16 (7.6)
Monthly patient volume	68 (40–100)
Surgeon availability*	200 (95.7)
Nuclear medicine availability*	144 (68.6)
MIP availability*	146 (69.5)
Availability of US in the endocrine department	98 (46.4)

* Responses to surgeon, nuclear medicine and MIT availability were, respectively, 209, 210 and 210.

IQR, interquartile range; NTD, nodular thyroid disease; MIP, minimally invasive procedures; US, ultrasound.

### Preferences for management in the standard case

**Figure 1:**
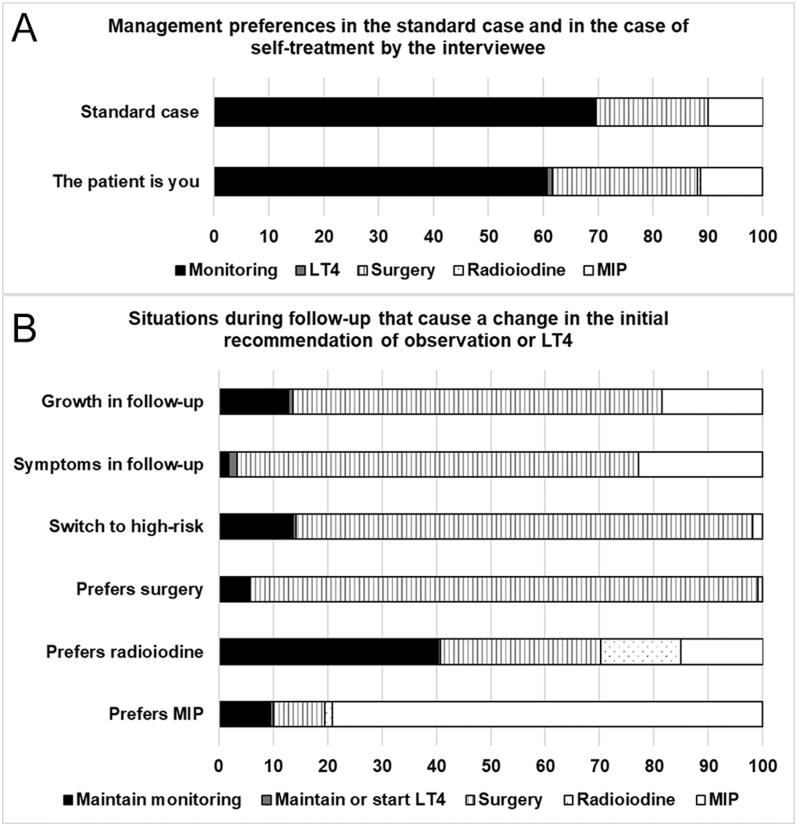
(A) Management preferences in the treatment of the standard case (a 50-year-old euthyroid asymptomatic woman with a single 4 cm benign thyroid nodule) and in the case where the patient is the interviewee. (B) Situations during the follow-up that induce a change in the attitude of endocrinologists in relation to the initial recommendation of monitoring or treatment with levothyroxine. Herein, the term ‘prefers’ refers to the patient’s preferences. Switch to high-risk refers to ultrasound features. LT4, levothyroxine; MIP, minimally invasive procedures.

In the standard patient management proposed in our survey, 147 (69.7%) endocrinologists indicated periodic monitoring as the procedure of choice, while 43 (20.4%) preferred surgical therapy and 21 (10.0%) preferred treatment by MIP (Fig. 1A). None opted for levothyroxine or radioiodine. These proportions were not different when endocrinologists were classified according to sex, age, years in practice, membership in international societies, private practice, monthly patient volume, surgeon availability and availability of US in the endocrine department (Table 2).

**Table 2:** Treatment preferences in the standard case* of the endocrinologists interviewed, classified according to demographic and professional characteristics. *P* values highlighted in bold indicate statistically significant differences.

Characteristics/group	*n*	Preferred management in standard case			*P*
		Monitoring	Surgery	MIP	
*n*	211	147	43	21	
Sex					
Female	131	91 (69.5)	28 (21.4)	12 (9.2)	0.824
Male	80	56 (70.0)	15 (18.8)	9 (11.3)	
Age, years					
<46	103	71 (68.9)	20 (19.4)	12 (11.7)	0.708
≥46	108	76 (70.4)	23 (21.3)	9 (8.3)	
Years in practice					
<20	108	76 (70.4)	20 (18.5)	12 (11.1)	0.708
≥20	103	71 (68.9)	23 (22.3)	9 (8.7)	
International membership					
No	149	107 (71.8)	28 (18.8)	14 (9.4)	0.571
Yes	62	40 (64.5)	15 (24.2)	7 (11.3)	
Private practice					
No	149	110 (73.8)	27 (18.1)	12 (8.1)	0.113
Yes	62	37 (59.7)	16 (25.8)	9 (14.5)	
% Patients with NTD					
≤30%	55	46 (83.9)	7 (12.7)	2 (3.6)	**0.027**
>30%	156	101 (64.7)	36 (23.1)	19 (12.2)	
Monthly patient volume					
<68	105	76 (72.4)	21 (20.0)	8 (7.6)	0.502
≥68	106	71 (67.0)	22 (20.8)	13 (12.3)	
Surgeon availability**					
No	9	5 (55.6)	1 (11.1)	3 (33.3)	0.057
Yes	200	140 (70.0)	42 (21.0)	18 (9.0)	
MIP availability**					
No	64	48 (75.0)	16 (25.0)	0 (0)	**0.005**
Yes	146	98 (67.1)	27 (18.5)	21 (14.4)	
US in endocrine department					
No	113	82 (72.6)	23 (20.4)	8 (7.1)	0.315
Yes	98	65 (66.3)	20 (20.4)	13 (13.3)	

NTD, nodular thyroid disease; MIP, minimally invasive procedures; US, ultrasound.

* The standard case is a 50-year-old woman with a single, solid, well-defined thyroid nodule measuring 4 cm, TIRADS 4 (intermediate risk, moderately suspicious). The cytological study is benign (Bethesda II). She does not present endothoracic extension. She is euthyroid and asymptomatic.

** There were two interviewees who did not respond to the question of surgeon availability and one who did not respond to the question of MIP availability.

However, physicians with a higher percentage of patients with BNTD in their clinical practice showed a greater preference for MIP (12.2 vs 3.6%) and a lower preference for monitoring (64.7 vs 83.9%; *P* = 0.027) compared to physicians with a lower percentage of patients with BNTD in their clinics (Table 2). As expected, endocrinologists with MIP availability also had a greater preference for this procedure compared to those without it (14.4% vs 0; *P* = 0.005). Chi-square analysis showed that there was no statistically significant relationship between the availability of MIP and the percentage of patients with nodular thyroid disease seen by the endocrinologist (*P* = 0.148).

### Variations in preferences

The variations in the preferences of the surveyed endocrinologists according to the characteristics of the nodule, the patient and the patient’s preferences are summarised in Table 3.

**Table 3:** Variations in management preferences according to different characteristics of nodular thyroid disease, patient and patient preferences. Data are presented as *n* (%) of responses for each management attitude of nodular thyroid disease in the proposed variations.

	Monitoring	Levothyroxine	Surgery	Radioiodine	MIP
Characteristics of nodular thyroid disease					
Nodular size 6 cm	19 (9.0)	1 (0.5)	164 (77.7)		27 (12.8)
Nodular size 3 cm	194 (91.9)		3 (1.4)		14 (6.6)
Dominant nodule in a MNG	143 (67.8)	1 (0.5)	60 (28.4)	2 (0.9)	5 (2.4)
Predominantly cystic nodule	103 (48.8)		5 (2.4)		103 (48.8)
Visible with the head in normal position	118 (55.9)		50 (23.7)		43 (20.4)
Asymptomatic endothoracic extension	126 (59.7)		76 (36.0)	1 (0.5)	8 (3.8)
Nodule incidentally detected PET-CT	108 (51.2)	3 (1.4)	96 (45.5)	1 (0.5)	3 (1.4)
Low risk, slightly suspicious (TIRADS 3)	178 (84.4)	1 (0.5)	20 (9.5)		12 (5.7)
High risk, very suspicious (TIRADS 5)	38 (18.0)	1 (0.5)	166 (78.7)	2 (0.9)	4 (1.8)
Demographic and clinical characteristics of the patient					
Age ≥65 years	162 (76.8)		27 (12.8)		22 (10.4)
Age ≤20 years	76 (36.0)	2 (0.9)	101 (47.9)		32 (15.2)
Male sex	118 (55.9)	1 (0.5)	77 (36.5)		15 (7.1)
Comorbidity that does not contraindicate surgery	148 (70.1)		42 (19.9)		21 (10.0)
Family history of non-medullary thyroid cancer	75 (35.5)		127 (60.2)		9 (4.3)
Head-neck radiation in childhood	54 (25.6)	1 (0.5)	148 (70.1)	1 (0.5)	7 (3.3)
Oncological disease	132 (62.6)	1 (0.5)	61 (28.9)	1 (0.5)	16 (7.6)
Patient preferences					
Declines any treatment	210 (99.5)				1 (0.5)
Prefers to take medication (levothyroxine)	169 (80.1)	13 (6.2)	23 (10.9)		6 (2.8)
Declines medication (levothyroxine)	174 (82.5)		19 (9.0)		18 (8.5)
Prefers surgery	18 (8.5)		188 (89.1)	1 (0.5)	4 (1.9)
Declines surgery	165 (78.2)	1 (0.5)	1 (0.5)		44 (20.9)
Is afraid of radioiodine	142 (67.3)		42 (19.9)		27 (12.8)
Prefers MIP	45 (32.3)		8 (3.8)	2 (0.9)	156 (73.9)
Declines MIP	149 (70.6)	3 (1.4)	55 (26.1)	1 (0.5)	3 (1.4)
Has difficulties attending follow-ups	48 (22.7)	1 (0.5)	132 (62.6)	5 (2.4)	25 (11.8)
Has cancerophobia	23 (10.9)		179 (84.8)	2 (0.9)	7 (3.3)
The patient is you	128 (60.7)	2 (0.9)	56 (26.5)		24 (11.4)

MIP, minimally invasive procedure; MNG, multinodular goitre, PET-CT, positron emission tomography/computed tomography; TIRADS, thyroid imaging reporting and data systems.

Changes in nodule size had an important impact on therapeutic decision-making. Comparing management preferences with the standard 4.0 cm nodule, the presence of a larger nodule (6.0 cm) shifted the preference in favour of surgery (from 20.4 to 77.7%). On the contrary, for smaller nodules (3.0 cm), the preferred recommendation was for monitoring (from 69.7 to 91.9%).

Imaging data and ultrasound features also had a significant impact on BNTD management. Again, compared with the standard case (TIRADS 4), the presence of a low-risk nodule (TIRADS 3) led to the preference for monitoring increasing from 69.7 to 84.4%. On the contrary, the preference for monitoring decreased to 18.0% in the high-risk nodule (TIRADS 5). Less markedly, monitoring decreased in the cystic nodule (48.8%), the visible nodule on physical exam (55.9%), the endothoracic extension nodule (59.7%) and the nodule detected in the PET-CT (51.2%).

At the same time, the preference for surgery increased in the US high-risk nodule (78.7%) and decreased in the cyst (2.4%) and the low-risk nodule (9.5%). The preference for MIP increased notably in nodules predominantly cystic (48.8%) and those visible on physical exam (20.4%) and was reduced in nodules detected on PET-CT (1.4%) and in high-risk nodules (1.8%).

The interviewees significantly reduced their preferences for monitoring and increased their preference for surgery in three groups of subjects: patients younger than 20 (monitoring: 36.0%; surgery: 47.9%), those with a family history of thyroid cancer (monitoring: 35.5%; surgery: 60.2%), and those with a past history of radiotherapy during childhood (monitoring: 25.6%; surgery: 70.1%). Interestingly, the preference for MIP increased in those younger than 20 (15.2%), but decreased in cases with a familial history of thyroid cancer (4.3%) or radiation during childhood (3.3%).

The choice for monitoring was clearly modified by the patient’s opinion: when preferring surgery or MIP, if there are difficulties with follow-ups or in cases of cancerophobia. In three of these four circumstances, the preference for surgery increased notably (surgery preferred: 89%; follow-up difficulties: 62.6%; cancerophobia: 84.8%), while it was reduced in the case of preference for MIP (3.8%).

The option for periodic observation increased in cases in which the patients refused treatment (99.5%), taking medication (82.5%) or when they preferred medical treatment with levothyroxine (80.1%). The advice for surgery was notably reduced in cases where the patient did not want it (0.5%). In line with the trend to follow the patient’s preferences, MIT increased very markedly when the patient desired it (73.9%), although this trend was reduced when the patient refused surgery (20.9%).

In all the included variants, the preference for levothyroxine or radioiodine was anecdotal, with the only exception of a 6.2% preference for levothyroxine when the patient preferred to take this medication.

The last question referred to how the specialist would self-treat in the case of having a thyroid nodule as the standard. A shift towards intervention was observed: 26.5% of the endocrinologists interviewed would opt for surgery and 11.4% for MIP, while monitoring drops to 60.7% (Fig. 1A).

### Recommendations for follow-up

**Figure 2:**
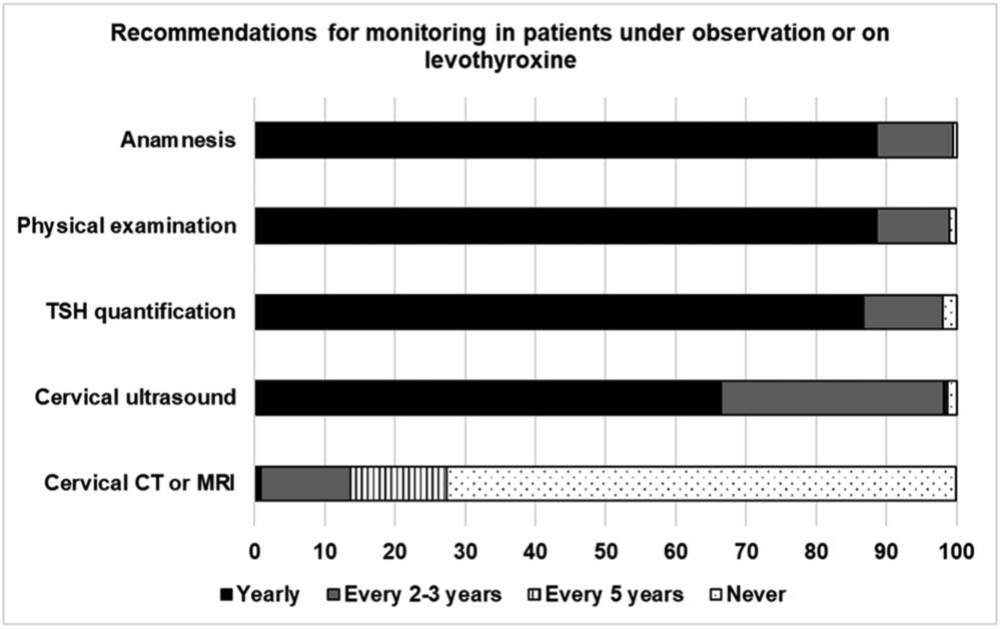
Frequency of complementary tests recommended by the interviewed endocrinologists during the follow-up of patients managed by periodic monitoring or treatment with levothyroxine. TSH, thyrotropin; CT, computed tomography; MRI, magnetic resonance imaging.

For patients under observation or on levothyroxine, most interviewees recommended an annual review, including anamnesis (88.6%), physical examination (88.6%) and serum TSH measurement (86.7%). Ultrasound was recommended annually by 66.4% and every 2–3 years by 31.8% endocrinologists. Cervical CT or MRI was rarely used (Fig. 2).

The clinical situations that forced specialists to change their initial recommendation of monitoring are depicted in Fig. 1B. A large part of specialists believed that surgery should be chosen if the patient presented during follow-up with any of the following: nodular growth (67.8%), symptoms (73.9%), switch to an ultrasound high-risk nodule (83.9%), or the patient’s preference for surgery (93.4%). Radioiodine was chosen by 14.7% interviewees when the patient prefers this therapeutic modality. Finally, 79.1% specialists changed their option to MIP when the patient prefers this procedure.

**Figure 3:**
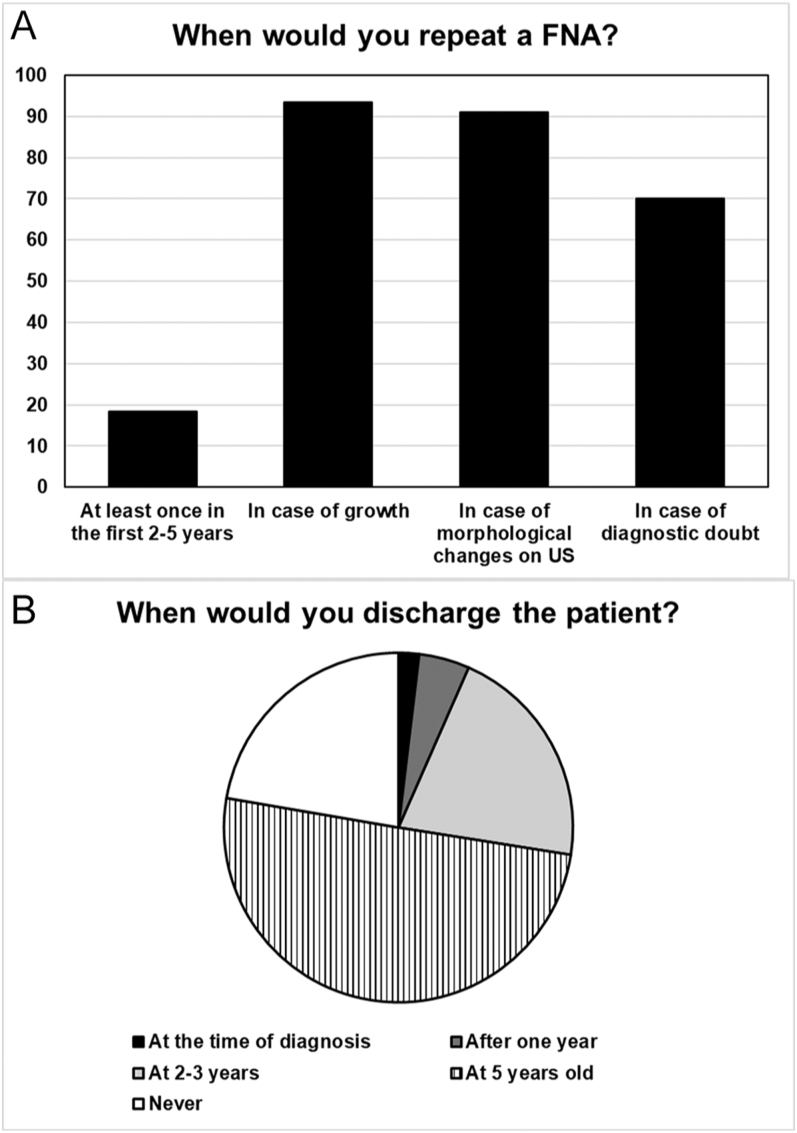
(A) Situations that arose during the follow-up of patients initially treated with observation or levothyroxine that, in the opinion of those interviewed, require a change in therapeutic attitude. (B) Opinion of the interviewees about the time to discharge the patient with nodular thyroid disease.

During follow-up, more than 90% respondents would repeat FNA in cases of nodular growth or morphological changes on US. Similarly, 70.1% would repeat this examination in cases of doubt with the first FNA and 18.5% would do it at least once (Fig. 3A). The study also showed that 20.9 and 50.2% interviewees believed that a stable patient should be discharged after 2–3 and 5 years, respectively. However, 22.3% specialists believed that patients should never be discharged (Fig. 3B).

## Discussion

In the past two decades, the management of BNTD has changed dramatically. The evaluation is no longer one-size-fits-all (2). During this time, new evidence has emerged that expands knowledge, opens up new therapeutic options and advises for a more conservative treatment. This change is well reflected in the constantly updated guidelines of the main international scientific societies, such as the American Thyroid Association (ATA) (4), the American Association of Clinical Endocrinologists (AACE) (5) and the European Thyroid Association (ETA) (6). However, little is known about how these improvements have been incorporated into the real-life management of this condition by specialists.

The usual recommendation for our index case (a 50-year-old euthyroid, asymptomatic woman with a single 4 cm benign thyroid nodule), according to these guidelines, would have been monitoring. Surgery may be considered in cases of growth, if the nodule causes compressive symptoms or upon clinical concern. As aforementioned, all these guidelines recommend against levothyroxine therapy.

**Figure 4:**
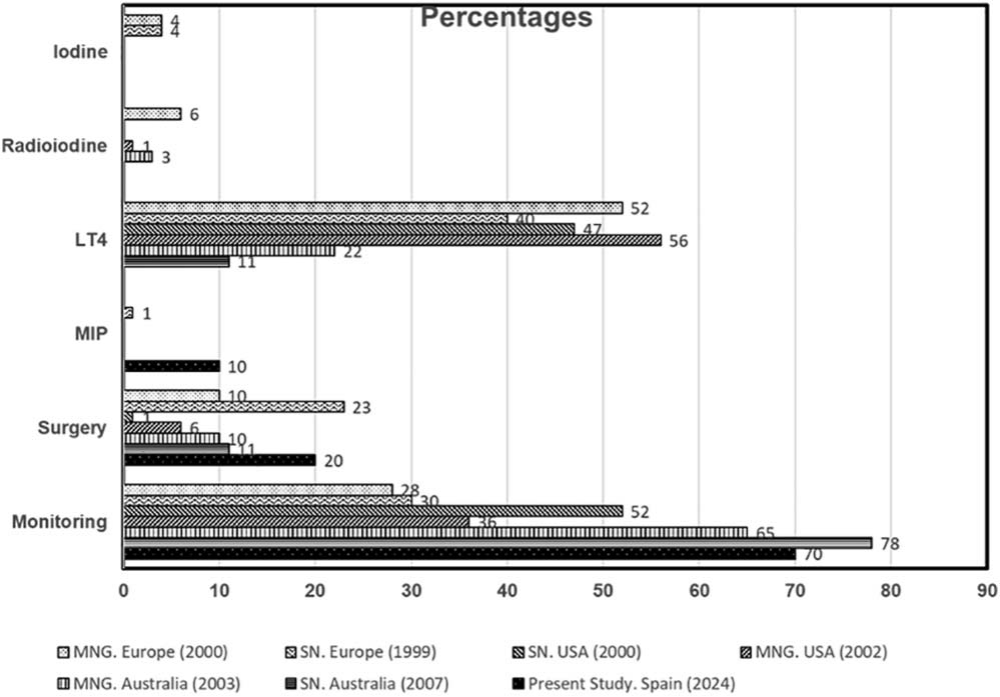
Summary of the results of similar surveys among endocrinologists from Europe, America and Australia (see text). LT4, levothyroxine; MIP, minimally invasive procedures.

Almost a quarter of a century before the publication of the latest ETA guidelines (6), a set of surveys comparable to ours reflected the strategies that were operational then throughout Europe (14, 15), the USA (16, 17) and Australia (18, 19) for the management of nodular thyroid disease. The Australian surveys focused on knowing the management differences between endocrinologists and endocrine surgeons, though. The surveys were carried out among members of international scientific societies interested in thyroid diseases (ETA and ATA). Participation, as usually occurs in this type of study, was moderate and practically anecdotal regarding the Spanish contributors. Surprisingly, the existence of large dissimilarities in the management of both nodular and simple goitre between the different continents of the Western world came to light. At that time, the most popular treatment option for euthyroid benign solitary thyroid nodule (SN) and multinodular goitre (MG) was levothyroxine: Europe: 40% (SN) and 52% (MG), and America: 47% (SN) and 56% (MG). Interestingly, American thyroidologists’ second option was surveillance: 52% (SN) and 36% (MG), while for the Australians, monitoring was always the first choice: 65% (MG) and 78% (SN). Wait and see also prevailed in the UK for the MNG (90%); for the rest of Europeans surveillance was chosen by 30% in cases of SN and 28% for MNG. Surgery was the third option in Europe: 10% (MG) and 23% (SN), whereas among American specialists, surgery was relegated to lower positions: 6% (MG) and 1% (SN). Radioactive iodine was selected for treatment by 6% (SN) European specialists (mainly from Denmark), unless in cases of suppressed serum TSH; in such cases, it was the main option for 44% European interviewees. In contrast, only 1% Americans chose radioactive treatment. MIP was almost completely unknown in those days; this explains why it was barely selected: in Europe, only 1% (SN) specialists bet for this option. The results of these surveys, along with present results, are depicted in Fig. 4.

With the limitations imposed by the fact that the previous studies are slightly different from the present one, it is nevertheless stimulating to discover how the international guidelines’ recommendations are echoed in medical practice, especially in a country (Spain) that does not have national guidelines for the management of BNTD. In this respect, the lack of national guidelines is probably an advantage, as specialists tend to look to international guidelines, which are often better documented.

From the present results, there are four aspects that deserve to be highlighted. First, treatment with levothyroxine has been gradually abandoned. This practice is strongly discouraged by international guidelines (4, 5, 6). None of the Spanish specialists considered this option in the present survey. Interestingly, in the 2022 previous Spanish survey within the THESIS project, 21.2% recommended this therapy for simple goitre (11). The possible explanation for this disagreement may be twofold: (i) THESIS results have already been published in the official Spanish Journal of Endocrinology and erroneous attitudes criticised; and (ii) the respondents to the present survey were probably more likely to be specialists with a strong interest in the management of thyroid diseases, while those interviewed in THESIS represented a broader sample of the endocrinologist community. In any case, it is good to see specialists now following best practices. Furthermore, it should be noted here that the THESIS study focused on the appropriate and inappropriate use of levothyroxine, whereas the present survey is specifically focused on the treatment of benign thyroid nodules, with five clearly defined therapeutic options. It is possible that this direct way of asking about the treatment of BNTD will allow more thoughtful responses to be obtained about the different treatments than a generic survey on possible uses of levothyroxine.

Second, the emergence of MIP in the therapeutic panorama of BNTD, with clear advantages over other options, has generated a significant number of followers. There is increasing evidence of its usefulness, as reflected by international guidelines (6, 8). Actually, the specific ETA guidelines, which are 8 years more up-to-date than the ATA ones, give greater emphasis on the indications of MIP (8). It should be noted that the indication for MIP based on the aspect of the nodule alone may be challenged in the absence of compressive symptoms, as it still is an invasive procedure, requires expertise and is still associated with a small risk of complications. Indeed, only 10% respondents opted for MIP in the standard case, while this percentage rose to 14.4% in endocrinologists who had MIP available in their hospital and to 22.7% in the case where the patient developed symptoms during follow-up. Unfortunately, MIP is not widely available in endocrinology clinics in our country, so it is reasonable to assume that it will gain greater relevance in the near future.

Interestingly, physicians who treat less than 30% patients with NTD preferred monitoring to a greater extent and MIP to a lesser extent than endocrinologists with a higher percentage of patients with thyroid disease. This could be due to the fact that the former are more conservative than the latter since we found no significant relationship between the availability of MIP and the percentage of patients treated with thyroid disease.

Third, the trend to prioritise active surveillance and monitoring over intervention in all nodular thyroid diseases (both benign and malignant) management is nowadays clearly supported by the guidelines (4, 5, 6). This implies a therapeutic de-escalation, with surgical options only if strictly necessary, and the withdrawal of radioactive iodine from the arsenal of BNTD management.

Finally, given that in many cases the therapeutic options are open, the guidelines increasingly place greater importance to the patient’s opinion (2, 6), an aspect that is essential to achieve good quality of care (20). Our results also reflect this trend since the choice between various therapies is more greatly influenced by the patient’s preferences. Today’s patients have a massive amount of information at their disposal and are readily able to discuss treatment options in an informed manner with their physician (21). Nevertheless, we must bear in mind that patient preferences are influenced by numerous factors that were not accounted for in our survey, such as the amount and type of information, the way in which it is presented, and even the wording used. In our survey, cancerophobia was the most influential factor shaping patient preferences, leading to a significant increase in preference for surgery and a decrease in the preference for monitoring. However, our survey did not allow the surveyed physicians to actively address this and other patient concerns. On the other side, the personal preference or bias of the physician may also be an important factor of variation, as reflected in the responses to our question ‘you are the patient’.

Our study is limited by its design within the field of endocrinology specialists in Spain; therefore, its results cannot be extrapolated to other geographical areas in Europe. Our survey was conducted among endocrinology specialists and did not include other specialists, as in Spain, most patients with BNTD are treated and followed up by an endocrinologist, regardless of whether the initial diagnosis was made by the primary care physician or by hospital specialists. The endocrinologist typically performs the diagnostic work-up and prescribes a treatment, while other specialists (surgeons or nuclear medicine physicians) are responsible for specific treatments when necessary. Long-term monitoring and possible treatment changes during follow-up are also carried out by endocrinologists. The dismal response rate of SEEN members (only 9%) is a limitation that certainly biases the results. In this type of study (surveys of specialists), there is a participation ceiling that is quite difficult to overcome because the effort of filling out a survey takes time and is not very personally rewarding. Sometimes, participation levels do not even reach 5% (22). The THESIS contribution was considered a great success with 26% participation (11). In a previous Spanish survey from 2020 on a related topic, only 12.4% participation was achieved, comparable to the current one (13).

In the current survey, we cannot know what fraction of the 211 respondents belonged to the SEEN Thyroid Task Force. Given that this Task Force is comprised of 124 members, it is clear that our survey generated interest beyond the Thyroid Task Force members, who are those with a specific academic interest in thyroid disease. This probably reflects the fact that thyroid pathology is highly prevalent and is a reason for consultation of most general endocrinologists. The present results, therefore, probably reflect the attitudes of those who have a real interest in thyroid diseases. In fact, from the content of the responses, it is probable that those who participated in the survey were specialists with a high dedication to thyroid diseases. Thus, we can infer that our results may mainly reflect the attitude of Spanish specialists highly committed to the practice of good thyroidology. Our survey considered surgery as a treatment option for BNTD but did not specify the type of surgery nor did it specifically inquire about the need for lifelong levothyroxine therapy after surgery. We also did not ask respondents whether they followed any particular clinical guidelines in their clinical practice.

In conclusion, the recommendations set forth in the international guidelines are having a positive impact on Spanish endocrinologists and represent a paradigm shift towards a more conservative management of BNTD: monitoring is prioritised as the option of choice, MIP is an increasingly popular option while surgery is reserved for highly suspicious or symptomatic cases. In addition, the use of levothyroxine for BNTD is falling into disuse. At the same time, specialists are giving significant weight to the patient’s opinion in therapeutic decision-making.

## Declaration of interest

The authors declare that there is no conflict of interest that could be perceived as prejudicing the impartiality of the work reported.

## Funding

The present investigation has not received any financial support from public sector agencies, commercial sector or non-profit entities.

## Patient consent

This study did not involve the participation or use of any patient data. The survey was conducted in an online format freely accessible to invited SEEN members. Since completion of the survey was voluntary, no written informed consent was considered necessary.

## Author contribution statement

The conceptualization, analysis and methodology were carried out by JJD and JCG. JJD and JCG also wrote the original draft. All authors have read and agreed to the published version of the manuscript.

## Data availability

Data are available through a reasonable request to the corresponding author.

## Ethics approval

This study received approval from the ethics committee of the Hospital Universitario Puerta de Hierro Majadahonda (code 57/899280.9/23).
